# Supplementary material for: Tracking W-Formate Dehydrogenase Structural Changes During Catalysis and Enzyme Reoxidation
Source: Int J Mol Sci. 2022 Dec 28;24(1):476. doi: 10.3390/ijms24010476 (PMC9820355; doi:10.3390/ijms24010476)
Supplement: Supplementary file 1 [file ijms-24-00476-s001.zip › ijms--supplementary_information.pdf]

Table S1- Data Collection and Refinement Statistics

| **Crystal** | **Fdh_1min** | **Fdh_1min-Staraniso** | **Fdh_1.5min** | **Fdh_1.5min-Staraniso** |
|---|---|---|---|---|
| **PDB$_{code}$** | | 8BQG | | 8BQH |
| **Diffraction Data** | | | | |
| Space group | $P2_12_12_1$ | $P2_12_12_1$ | $P2_12_12_1$ | $P2_12_12_1$ |
| Cell dimensions (Å) (º) | a=64.54, b=122.78, c=148.99 | a=64.54, b=122.78, c=148.99 | a=64.36, b=127.46, c=128.95 | a=64.36, b=127.46, c=128.95 |
| Wavelength (Å) | 0.8856 | 0.8856 | 0. 8856 | 0. 8856 |
| Beamline | ESRF ID23-1 | ESRF ID23-1 | ESRF ID23-1 | ESRF ID23-1 |
| No. Crystals | 1 | 1 | 1 | 1 |
| Resolution range of data (Å) (last shell) | 94.75 – 2.19 (2.23 – 2.19) | 94.75 – 1.95 (2.07 – 1.95) | 90.65 – 1.90 (1.93 – 1.90) | 90.65 – 1.61 (1.78 – 1.61) |
| Completeness (%) (last shell) | 97.72 (99.22) | 91.96 (46.32) | 99.35 (100.00) | 94.17 (64.00) |
| Rmerge (last shell) | 0.090 (0.709) | 0.102 (1.055) | 0.120 (0.803) | 0.130 (0.877) |
| Rmeas (last shell) | 0.102(0.808) | 0.116 (1.212) | 0.132 (0.883) | 0.143 (0.989) |
| I/σI (last shell) | 10.9 (2.1) | 9.2 (1.5) | 10.4 (2.2) | 9.5 (1.7) |
| CC 1/2 (last shell) | 0.997 (0.704) | 0.997 (0.435) | 0.996 (0.779) | 0.996 (0.656) |
| Redundancy (last shell) | 4.3 (4.2) | 4.3 (4.1) | 5.6 (5.8) | 5.7 (4.7) |
| | | | | |
| **Refinement** | | | | |
| Reflections used in refinement (work (free)) | | 69752 (3564) | | 90038 (4778) |
| Rwork | | 0.193 | | 0.151 |
| Rfree | | 0.243 | | 0.175 |
| Nº of non-hydrogen atoms | | 9509 | | 9927 |
| Protein | | 9191 | | 9134 |
| Ligands | | 168 | | 229 |
| Ions | | 2 | | 7 |
| Solvent | | 148 | | 557 |
| **Geometry and *B*-factors** | | | | |
| RMSD bond lengths (Å) | | 0.010 | | 0.012 |
| RMSD bond angles (º) | | 1.618 | | 1.601 |
| Average B-factor ALL (Å$^2$) | | 38.30 | | 19.36 |
| Protein (Å$^2$) | | 40.86 | | 20.06 |
| Ligands (Å$^2$) | | 34.75 | | 22.61 |
| Ions (Å$^2$) | | 32.68 | | 31.75 |
| Solvent (Å$^2$) | | 34.24 | | 25.26 |
| Ramachandran favoured (%) | | 95.72 | | 96.98 |
| Ramachandran outliers (%) | | 0.26 | | 1.04 |
| Molprobity score | | 1.87 | | 1.31 |
| Clashscore | | 4.77 | | 3.30 |

| **Crystal** | **Fdh_3min** | **Fdh_5min** | **Fdh_5min-Staraniso** |
|---|---|---|---|
| **PDB$_{code}$** | 8BQI | | 8BQJ |
| **Diffraction Data** | | | |
| Space group | $P2_12_12_1$ | $P2_12_12_1$ | $P2_12_12_1$ |
| Cell dimensions (Å) (º) | a=64.99, b=123.75, c=150.36 | a=64.94, b=123.35, c=150.55 | a=64.94, b=123.35, c=150.55 |
| Wavelength (Å) | 0. 8856 | 0. 8856 | 0. 8856 |
| Beamline | ESRF ID23-1 | ESRF ID23-1 | ESRF ID23-1 |
| No. Crystals | 1 | 1 | 1 |
| Resolution range of data (Å) (last shell) | 150.36 – 2.36 (2.43 – 2.36) | 95.41 – 2.58 (2.62 – 2.58) | 95.41 – 2.11 (2.36 – 2.11) |
| Completeness (%) (last shell) | 98.30 (91.50) | 99.08 (99.69) | 92.88 (62.30) |
| Rmerge (last shell) | 0.139 (1.258) | 0.110 (0.806) | 0.121 (0.851) |
| Rmeas (last shell) | 0.170 (1.524) | 0.127 (0.927) | 0.139 (0.978) |
| I/σI (last shell) | 9.0 (1.2) | 10.4 (2.2) | 9.1 (1.8) |
| CC 1/2 (last shell) | 0.992 (0.409) | 0.994 (0.621) | 0.994 (0.552) |
| Redundancy (last shell) | 4.8 (4.8) | 4.3 (4.2) | 4.3 (3.9) |
| | | | |
| **Refinement** | | | |
| Reflections used in refinement (work (free)) | 47534 (2409) | | 43543 (2296) |
| Rwork | 0.213 | | 0.214 |
| Rfree | 0.248 | | 0.245 |
| Nº of non-hydrogen atoms | 9442 | | 9431 |
| Protein | 9201 | | 9191 |
| Ligands | 174 | | 159 |
| Ions | 2 | | 2 |
| Solvent | 65 | | 79 |
| **Geometry and *B*-factors** | | | |
| RMSD bond lengths (Å) | 0.003 | | 0.003 |
| RMSD bond angles (º) | 1.282 | | 1.234 |
| Average B-factor ALL (Å$^2$) | 44.55 | | 38.78 |
| Protein (Å$^2$) | 47.73 | | 41.62 |
| Ligands (Å$^2$) | 40.83 | | 33.80 |
| Ions (Å$^2$) | 37.43 | | 31.59 |
| Solvent (Å$^2$) | 39.63 | | 29.84 |
| Ramachandran favoured (%) | 94.78 | | 95.21 |
| Ramachandran outliers (%) | 0.34 | | 0.43 |
| Molprobity score | 1.67 | | 1.54 |
| Clashscore | 2.54 | | 3.42 |

| **Crystal** | **Fdh_22min** | **Fdh_22-Staraniso** | **Fdh_FormO2** | **Fdh_FormO2-Staraniso** |
|---|---|---|---|---|
| **PDB$_{code}$** | | 8BQK | | 8BQL |
| **Diffraction Data** | | | | |
| Space group | $P2_12_12_1$ | $P2_12_12_1$ | $P2_12_12_1$ | $P2_12_12_1$ |
| Cell dimensions (Å) (º) | a=64.58, b=123.87, c=149.18 | a=64.58, b=123.87, c=149.18 | a=64.80, b=127.59, c=148.62 | a=64.80, b=127.59, c=148.62 |
| Wavelength (Å) | 0.7749 | 0.7749 | 0.9686 | 0.9686 |
| Beamline | ESRF ID23-1 | ESRF ID23-1 | ESRF ID30B | ESRF ID30B |
| No. Crystals | 1 | 1 | 1 | 1 |
| Resolution range of data (Å) (last shell) | 95.30 – 1.80 (1.83 – 1.80) | 95.30 – 1.59 (1.70 – 1.59) | 96.81 – 2.11 (2.15 – 2.11) | 96.81 – 1.91 (2.01 – 1.91) |
| Completeness (%) (last shell) | 99.13 (99.24) | 92.25 (63.11) | 99.37 (99.89) | 94.46 (61.60) |
| Rmerge (last shell) | 0.070 (0.656) | 0.077 (1.125) | 0.168 (1.088) | 0.186 (1.686) |
| Rmeas (last shell) | 0.079 (0.745) | 0.040 (1.234) | 0.177 (1.142) | 0.195 (1.767) |
| I/σI (last shell) | 13.0 (2.2) | 11.3 (1.5) | 10.2 (2.2) | 9.1 (1.3) |
| CC 1/2 (last shell) | 0.998 (0.670) | 0.998 (0.579) | 0.997 (0.737) | 0.997 (0.620) |
| Redundancy (last shell) | 4.5 (4.5) | 4.7 (5.9) | 10.7 (11.1) | 11.0 (11.1) |
| | | | | |
| **Refinement** | | | | |
| Reflections used in refinement (work (free)) | | 123277 (6509) | | 78325 (4045) |
| Rwork | | 0.170 | | 0.188 |
| Rfree | | 0.192 | | 0.225 |
| Nº of non-hydrogen atoms | | 9875 | | 9769 |
| Protein | | 9219 | | 9228 |
| Ligands | | 196 | | 166 |
| Ions | | 2 | | 2 |
| Solvent | | 458 | | 373 |
| **Geometry and *B*-factors** | | | | |
| RMSD bond lengths (Å) | | 0.007 | | 0.004 |
| RMSD bond angles (º) | | 1.387 | | 1.278 |
| Average B-factor ALL (Å$^2$) | | 28.48 | | 33.79 |
| Protein (Å$^2$) | | 30.01 | | 36.02 |
| Ligands (Å$^2$) | | 29.19 | | 28.03 |
| Ions (Å$^2$) | | 23.15 | | 25.68 |
| Solvent (Å$^2$) | | 33.81 | | 31.61 |
| Ramachandran favoured (%) | | 96.32 | | 96.67 |
| Ramachandran outliers (%) | | 0.26 | | 0.34 |
| Molprobity score | | 1.35 | | 1.18 |
| Clashscore | | 3.12 | | 2.00 |

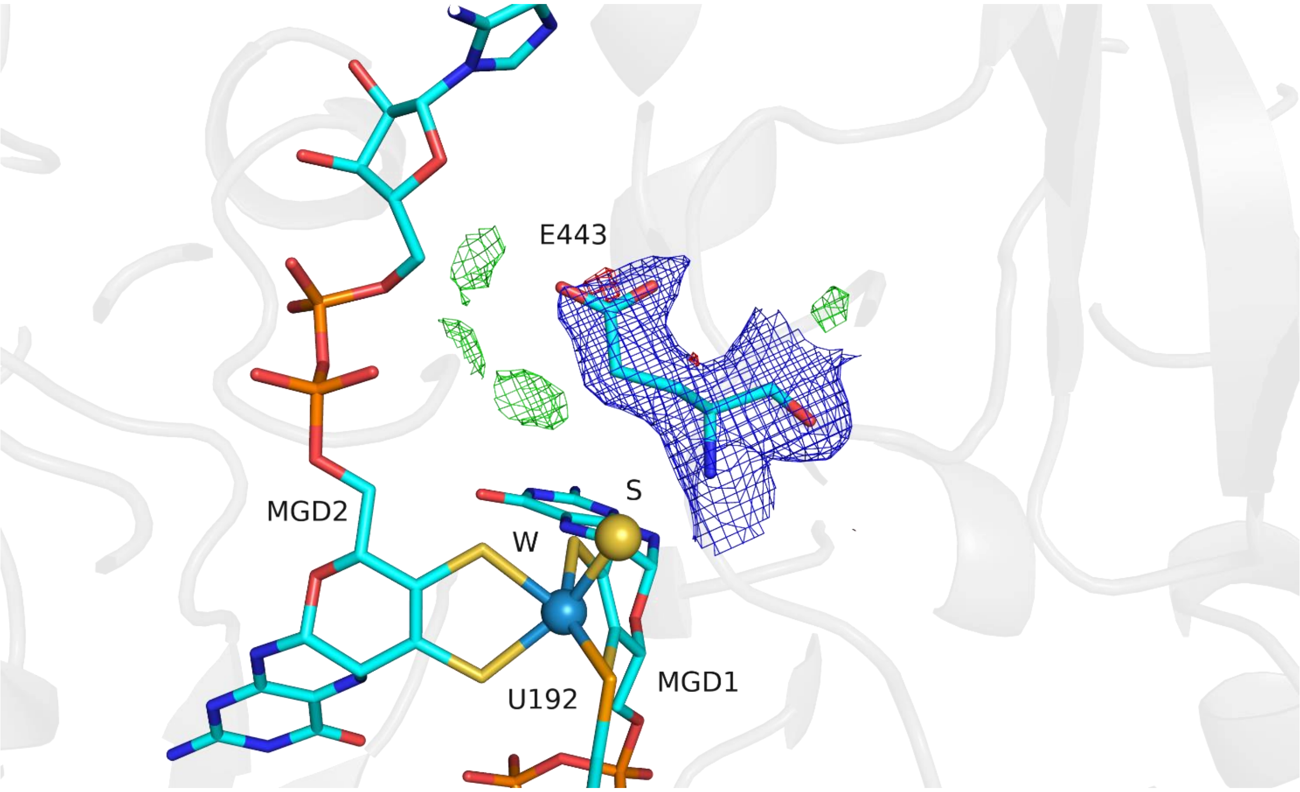


**Figure S1.** *Dv*FdhAB formate-soaked Fdh_5min structure (light blue). U192, E443 and the two MGD co-factors are shown as sticks. The 2fo-fc map is shown as a blue mesh, at 1.0 σ, and the fo-fc map is shown as a green and red mesh, respectively, for positive and negative values, at 3.0 σ.
